# Brainstem auditory evoked responses: Objective hearing threshold assessment in Holstein cows

**DOI:** 10.4102/sajcd.v71i1.1047

**Published:** 2024-10-21

**Authors:** Alida Naudé, Lize-Mari Erasmus, Liesl de Swardt, Juan Bornman, Este van Marlé-Köster

**Affiliations:** 1 Centre for Augmentative and Alternative Communication (CAAC), Faculty of Humanities, University of Pretoria, Pretoria, South Africa; 2 Department of Animal Science, Faculty of Natural and Agricultural Sciences, University of Pretoria, Pretoria, South Africa; 3 Faerie Glen Animal Clinic, Pretoria, South Africa; 4 Department of Speech-Language and Hearing Therapy, Faculty of Medicine and Health Sciences, Stellenbosch University, Stellenbosch, South Africa

**Keywords:** absolute wave latencies, animal audiology, auditory sensitivity, BAER, cattle, Holsteins cows, interpeak latencies

## Abstract

**Background:**

Animal audiology utilizes brainstem auditory evoked responses (BAER) as a non-invasive tool to assess hearing in animals, including Holstein dairy cows. Understanding cows’ auditory capabilities is critical for their welfare, especially given their exposure to farm noise.

**Objectives:**

This study provides preliminary normative BAER data for Holstein cows by focusing on absolute and interpeak wave latencies. The objective is to assess the impact of farm noise and expand audiologists’ practice scope.

**Method:**

Ten Holstein cows were tested using monoaural broadband click stimuli with contralateral masking. Earphones with foam ear tips were used to minimize environmental noise interference. The BAER responses were recorded via subdermal needle electrodes placed at standardized locations on the cows’ heads. The data were analysed using descriptive statistics to determine auditory thresholds and wave latencies.

**Results:**

The cows exhibited auditory thresholds at 90 dB SPL (55 dB nHL). Detailed wave and interpeak latencies were recorded at intensities from 85 to 105 dB SPL. At 90 dB SPL, the average latency for wave V was 5.17 ms, marking the auditory threshold for Holstein cows.

**Conclusion:**

These findings provide key insights into the auditory sensitivity of Holstein cows, highlighting BAER’s potential for monitoring auditory health and evaluating the effects of noise pollution on animal welfare. This research underscores the value of integrating animal audiology into the audiologist’s scope, ultimately enhancing both animal welfare and farming sustainability.

**Contribution:**

This study adds to the limited literature on farm animal auditory health and suggests strategies to improve welfare through better auditory management.

## Introduction

In animal audiology, brainstem auditory evoked responses (BAER) can be used as a reliable method to determine hearing thresholds, serving a critical role in diagnosing various forms of deafness across species. Cows, integral to dairy farming, interact with their environment using all five senses, with hearing playing a pivotal role in their safety, comfort and overall welfare. The way cows perceive and evaluate their environment is significantly influenced by their auditory capabilities and has direct implications for their behaviour, stress levels and productivity on farms (Veissier & Boissy, 2007). Cows have a natural reflex for listening, frequently pricking their ears to detect sounds. Their highly developed auditory abilities enable them to rotate their ears at the base of their heads, helping them identify sounds from all directions (Heffner, 1998). Their ability to hear and respond to their surroundings is closely connected to their basic biological needs for comfort, which include safety, fear, flight behaviour and aggression (Doyle & Moran, 2015).

Vocalisations, referred to as auditory communication, are an integral part of their communication with each other (Green et al., 2019). A study by Heffner (1998) has shown that calves are able to recognise their mother’s calls. Auditory input is essential for vocal learning (Sewall, 2012), and they also respond to the vocalisations produced by other species (Phillips, 2018). Moreover, research has shown that vocalisations can be an indicator of stress in cattle (Brouček, 2014). Vocalisations thus play a role in communication and emotional expression, both of which are important indicators of farm animal welfare. Despite the obvious benefits of measuring auditory thresholds and applying auditory stimuli like music in farm environments (Lemcke et al., 2021; Waiblinger, 2019), there is a nascent body of research on the effects of environmental noise, on farm animals such as cows. Research has shown that music can have a positive impact on dairy cows, particularly in terms of milk production. For example, studies have demonstrated that certain types of music, when played in the milking parlour, can reduce stress levels in cows, leading to an increase in milk yield (Lemcke et al., 2021). This effect is thought to be because of the calming influence of rhythmic and melodic sounds, which can help regulate the cows’ stress responses during milking.

Additionally, consistent exposure to music in automated milking systems has been associated with more frequent and efficient milking intervals, further contributing to overall productivity (Waiblinger, 2019). These findings suggest that the auditory environment plays a significant role in cow welfare and productivity, reinforcing the importance of further research in this area to optimise farm management practices and enhance animal well-being.

This increasing research interest in the impact of environmental noise on dairy cows such as Holsteins is possibly linked to the increasing public concern for the welfare of farm animals (Laurijs et al., 2021). Emerging evidence shows that noise levels on dairy farms can significantly affect the well-being and productivity of cows (Dimov et al., 2023; Pšenka et al., 2016). Studies have reported that noise levels in dairies can vary widely depending on the type of equipment used, the layout of the facilities and the management practices in place. For example, Brouček (2014) found that the noise from machinery, such as milking machines and tractors, can reach levels as high as 97 dB, which is above the threshold that can cause stress and discomfort in cows. Similarly, noise levels in milking parlours have been measured at around 72.5 dB, with automated systems sometimes producing lower levels at around 67.9 dB (Pšenka et al., 2016). These findings highlight the potential for chronic exposure to high noise levels in dairy environments to impact the welfare of dairy cows negatively, particularly in terms of auditory stress and its subsequent effects on behaviour and milk production. The present study aims to determine preliminary data to establish normative BAER data for Holstein dairy cows and to assess how environmental noise may influence their auditory thresholds. This information may provide a foundation for understanding the broader implications of noise exposure on the welfare of dairy cows such as Holsteins.

The hearing system of Holstein cows is quite similar to that of humans, especially in how sound travels from the ear to the brain. However, there are also notable differences in anatomy and physiology between the two species. In cows, the auricle is larger and more mobile than the human pinna, allowing the auricle to better capture and funnel sound waves into the ear canal. Once the sound waves enter the ear canal, they follow a pathway like that of humans: the vibrations of the tympanic membrane are transmitted through the ossicles (malleus, incus and stapes) to the cochlea in the inner ear (Gelfand, 2016).

Within the cochlea, mechanical vibrations are converted into electrical signals by the hair cells. These signals are then transmitted via the auditory nerve to the brainstem and further processed in the auditory cortex, like the human auditory system. The auditory pathway in cows involves peripheral structures (outer, middle and inner ear) and central auditory processing regions, including the cochlear nuclei, superior olivary complex, lateral lemniscus, inferior colliculus, medial geniculate nuclei and auditory cortex, which are also present in humans (Peterson et al., 2020). However, cows may have different frequency sensitivity ranges compared to humans, which are adapted to their specific environmental needs and communication patterns. Despite these anatomical differences, the fundamental process of sound transmission and neural processing in cows remains consistent with the general principles observed in the human auditory system.

Hearing ability and auditory threshold (the lowest sound audible) are expressed in terms of the loudness of sound, measured in decibels (dB), as well as the frequency of sound, measured in hertz (Hz). In terms of frequency, a cow’s hearing ranges from 23 Hz to 37 000 Hz (Heffner, 1998), which implies that they perceive a larger range of frequencies compared to the human ear (Delpietro, 1989). According to Dorleáns (2019), cows have a sound threshold of 85 dB – 90 dB sound pressure level (SPL) and any range of environmental sounds exceeding 110 dB SPL may cause physical damage (Phillips, 2018; Pšenka et al., 2016). Dairy breeds are reported to have superior responses to auditory stimulation when compared to beef breeds (Lanier et al., 2000). Hearing is determined genetically but animal factors such as physiological state, breed and age also affect the way sound is perceived (Brouček, 2014).

Brainstem auditory evoked responses is an electrodiagnostic test used to estimate auditory thresholds by measuring the electrical potentials evoked from an animal’s auditory system. This non-invasive and objective procedure involves delivering a multi-tonal click stimulus to the test ear vial earphones allowing for the recording of bio-electrical potentials from auditory pathway structures. These potentials can be registered within 10 ms following a brief auditory stimulus (Legatt, 2013).

The use of BAER has become an increasingly valuable tool in assessing auditory function in livestock, particularly in cattle. This non-invasive technique allows for the measurement of auditory thresholds and neural conduction times, offering insights into the auditory health of animals in various environments. Previous studies, such as those by Uetake et al. (1996) and Strain et al. (1989), were instrumental in establishing baseline auditory thresholds in cattle using normalised hearing levels (nHL). Uetake et al. (1996) found that behavioural thresholds to pure tones ranged between 20 and 45 dB SPL in the high-frequency range (2000 Hz – 4000 Hz), corresponding to approximately 55 dB nHL in BAER assessments. Similarly, Strain et al. (1989) reported auditory thresholds of 65 dB–75 dB nHL, highlighting the lower hearing thresholds in cattle compared to other livestock, such as horses.

Arai and Matsui (2008) further validated the use of BAER in cattle, providing additional evidence of its effectiveness in assessing auditory function. These foundational studies have set the stage for continued research in this area, emphasising the importance of developing reliable methods for monitoring auditory health in cattle, particularly in noisy farm environments.

Building on this body of research, the current study aims to provide updated normative data on BAER thresholds and latencies in Holstein cows. By comparing our findings with those of previous studies, we seek to contribute to the evolving field of animal audiology and enhance our understanding of how environmental factors may impact the auditory health of dairy cattle.

Gonzalez et al. (2019) conducted a pivotal study developing a protocol for obtaining BAER in Holstein calves and establishing normal parameters for peak latencies, interpeak latencies (IPL) and waveforms in this species. Their research demonstrated that BAER is a minimally invasive method suitable for on-farm applications, allowing for the selection of animals with optimal hearing conditions. The study found that recorded peak latencies and IPLs were consistent with previous studies on adult male bulls, indicating the reliability of BAER across different age groups within the species.

The findings by Gonzalez et al. (2019) underscore the utility of BAER in assessing auditory thresholds and monitoring auditory function in cattle, which is crucial for early detection of hearing impairments that could affect animal welfare and productivity. This aligns with the growing body of literature supporting the use of BAER in veterinary medicine, particularly for its ability to differentiate between various types of hearing loss, including pre-cochlear, cochlear and central auditory impairments.

The BAER test does not require any voluntary response from the subject, making it ideal for estimating auditory thresholds in animals (Arai & Matsui, 2008). It provides a summation of electrical activity generated by various structures within the auditory system in response to repeated acoustic stimuli. These measurements are taken at different loudness levels across frequencies, enabling the evaluation of the auditory pathway up to the level of the brainstem. The maximum amplitude of the BAER can be recorded from different cranial points, with the highest amplitude typically obtained from an electrode at the vertex, referenced to the stimulated ear (Gonzalez-Blanco et al., 2019).

A limited number of studies have reported hearing thresholds in Holstein cows using BAER. In a study by Strain et al. (1989), BAER thresholds between 65 dB and 75 dB normalised hearing level (nHL) were detected in 29 cows, which were found to be higher compared to those in horses. A similar range at 65 dB–75 dB nHL was reported for 10 Holstein cows and Japanese Black cattle using BAER (Arai & Matsui, 2008), while Uetake et al. (1996) performed a frequency-specific BAER assessment in three Holstein calves obtaining average BAER thresholds between 54 dB and 90.7 dB nHL for frequencies 500 Hz–8000 Hz.

Brainstem auditory evoked responses has thus proven useful in evaluating auditory function in animals because of its objectivity, reliability, relatively low cost, time effectiveness and non-invasiveness (Wilson & Mills, 2005). The application of BAER normative data has proven useful in studying various bovine conditions, including bovine spongiform encephalopathy (Arai & Matsui, 2008) and genetic disorders involving the Coch gene (Ikezono et al., 2001) as well as pigmentation-related conditions (Philipp et al., 2011).

In this study, the Holstein cows selected for BAER testing displayed normal pigmentation, characterised by the typical black and white coat patterns, which are associated with certain genetic traits. The importance of pigmentation lies in its link to genetic disorders, such as congenital sensorineural deafness, which has been observed in cattle with certain pigmentation patterns, similar to conditions seen in other species. The MITF gene, which influences pigmentation, has been associated with hearing impairments in various animals, including domestic animals (Strain, 2015) and cattle (Petersen et al., 2023; Philipp et al., 2011) suggesting that pigmentation patterns in cows might also correlate with auditory health risks.

By establishing normative BAER data for cows with typical pigmentation patterns, this study contributes valuable information that could be used in future research to better understand the potential genetic links between pigmentation and hearing disorders in cattle. This connection underscores the importance of considering pigmentation as a factor in auditory health assessments, particularly in dairy breeds like Holsteins.

The expansion of audiology into the animal domain offers a unique opportunity to enhance the welfare and management of farm animals, particularly dairy cows, which are subjected to a range of farm noises daily. However, it is important to acknowledge that, within the South African context, animal audiology is not recognised by the Health Professions Council of South Africa to be within the scope of practice for audiologists. Typically, veterinarians perform hearing screenings, such as pass or refer assessments, without determining auditory thresholds. However, audiologists who have received specialised training in animal audiology are qualified to conduct diagnostic tests, which include neurological assessment as well as threshold determination. This specialised role allows trained audiologists to make significant contributions to animal welfare through more detailed and accurate auditory assessments than merely hearing screening. By understanding the auditory capabilities and susceptibilities of animals, in this case, dairy cows, audiologists can contribute to the development of better farm management practices that consider the auditory comfort of cows, potentially leading to improved milk production and overall welfare.

Furthermore, the inclusion of animal audiology within the audiologist’s scope of practice enriches the field by opening new avenues for research, practice and interdisciplinary collaboration. This not only broadens the understanding of auditory health and function across species but also contributes to the development of innovative auditory care strategies that benefit a wide range of animals. Using BAER to assess the hearing thresholds of Holstein cows demonstrates the significant insights that animal audiology can offer regarding the impact of environmental noise on farm animals and the potential to improve their welfare through better auditory health management.

Thus, this study aims to provide preliminary data for establishing normal parameters of BAER in Holstein cows, offering a foundation for future research in animal audiology and contributing to the broader scope of practice for audiologists. By exploring the auditory capabilities of dairy cows, this research highlights the significance of auditory health in animal welfare and the critical role of audiologists in advancing the well-being of animals in addition to that of humans. This study serves as the first part of a bigger welfare study, conducted by Erasmus et al. (2023).

This study is particularly relevant to the South African context, where the agricultural sector plays a vital role in the economy and food security. Dairy farming is a significant part of this sector, and ensuring the welfare and productivity of dairy cows is essential for sustainable farming practices (Erasmus et al., 2023). The application of BAER in assessing the auditory health of Holstein cows is not only innovative but also aligns with the increasing focus on animal welfare and management in South Africa.

In the South African setting, where environmental noise from farming operations can vary significantly and affect animal welfare, this study offers essential insights into monitoring and managing auditory health. Noise is regarded as a major factor affecting the welfare of dairy cows, but the severity of it is often underestimated or ignored (Cecchini et al., 2024). Therefore, by exploring the auditory thresholds of dairy cows using BAER, the study opens new possibilities for integrating advanced veterinary audiological practices into South African dairy farming, which could lead to improved animal welfare and produstivity. The study also highlights the need for specialised training for South African audiologists who wish to expand their scope of practice to include animal audiology, a field that holds great potential in the country’s agricultural landscape.

The potential of animal audiology, particularly using BAER, extends beyond the immediate benefits to animal welfare and productivity. Farm animal welfare is closely tied to the sustainable development goals (SDGs), highlighting the profound link between human advancement and the compassionate treatment of animals. Keeling et al. (2019) stated that animal welfare forms a vital part of the success of every single SDG. Production animals, including dairy cows, are essential in achieving the aim set forth by the United Nations in their SDGs (Olmos Antillón et al., 2021). To achieve these goals, efficient production is essential – which goes hand in hand with sound animal welfare (Sebo et al., 2022). As such, animal welfare is aligned with particularly SDG 1 (No poverty), SDG 2 (Zero Hunger), SDG 3 (Good Health and Well-being) and SDG 12 (Responsible Consumption and Production) (United Nations, 2015).

Sustainable development goals 1 and 2 emphasise the need to end hunger and ensure sustainable food production systems. By improving the welfare and management of dairy cows through precise auditory health assessments, animal audiology contributes to enhanced milk production and overall farm productivity, supporting food security.

Sustainable development goal 3 focusses on ensuring healthy lives and promoting well-being for all. Healthier livestock means healthier people. Animal audiology enhances the well-being of dairy cows by identifying and mitigating the impacts of environmental noise, which can lead to stress and reduced productivity. By safeguarding the auditory health of these animals, the field contributes to the broader One Health initiative, which recognises the interconnected well-being of animals, humans and the environment.

Sustainable development goal 12 encourages sustainable consumption and production patterns. The integration of advanced diagnostic tools like BAER in dairy farming promotes responsible production by ensuring that animals are maintained in environments that minimise stress and maximise productivity, ultimately leading to more sustainable farming practices.

By linking the practice of animal audiology to these global goals, this study highlights the broader impact of ensuring the auditory health and welfare of dairy cows. It underscores the value of incorporating such practices into routine veterinary care and farm management, not only to benefit the animals but also to contribute to global efforts towards sustainability and responsible farming.

## Research methods and design

### Research design

This study utilised an experimental analytical quantitative research design to provide preliminary data for establishing normative BAER data for Holstein cows. The research aimed to assess their auditory thresholds and sensitivity, with a focus on understanding how environmental noise prevalent in dairy farm environments may influence the auditory health and welfare of these animals. Normative data describe values that are usual in a group and allow for comparison to subsequent values. The study was approved by the institutional ethics committee of the University of Pretoria’s Faculty of Veterinary Science, specifically the Animal Ethics Committee, under AEC number NAS341/2020.

### Setting

The study was conducted at the Hillcrest experimental farm of the University of Pretoria. The farm employs a zero-grazing system, where cows are housed in an open dirt lot and are exposed to various noises such as tractors, feed wagons and milking machines during milking three times per day. The relevance of this setting is that the zero-grazing system ensures continuous exposure to consistent noise levels, which could have a cumulative effect on the cow’s auditory health. Understanding the impact of these noise exposures is crucial for assessing potential stress and auditory strain on the cows.

### Sample and inclusion criteria

The study involved 10 Holstein cows (20 ears), aged 42.6 months ± 7.2 months (mean ± standard deviation), all within their second lactation. The selection of cows in their second lactation was based on their stable physiological and reproductive status, which ensures reduced variability in auditory responses that could be influenced by age-related factors. Second-lactation cows are generally in peak production, making them an ideal group for studying the impact of environmental factors, such as noise, on auditory health and overall welfare.

Our methodology mirrored the approach of Gonzalez-Blanco et al. (2019), where the study included a thorough physical examination of the ear canals, focussing on identifying any malformations, secretions or other abnormalities. Animals displayed normal pigmentation of their coats and eye colour. Any animal exhibiting signs of systemic illness, visible ear canal disorders or a lack of response to environmental sound stimuli was excluded from the study. Neither food nor water was withheld before BAER testing to maintain the cows in a normal, unstressed state, ensuring that the measurements accurately reflected their baseline auditory function without the confounding influence of invasive and disrupting factors causing distress.

### Procedure

Brainstem auditory evoked responses recordings were performed on all cows while they were in a standing position, with each cow’s head securely restrained using a guillotine head gate. An experienced farm animal handler, familiar with the cows and responsible for their daily care, assisted in providing additional restraint using a halter when necessary. Importantly, no pharmacological agents or nose tongs were used to ensure restraint.

Two audiologists were integral members of the testing team, ensuring that the procedures were conducted according to audiological standards and best practices. The BAER setup included a minimum of three electrodes: a recording electrode, a ground electrode and a reference electrode. This setup also required an electrode board, amplifier, signal averager and stimulator.

**FIGURE 1:**
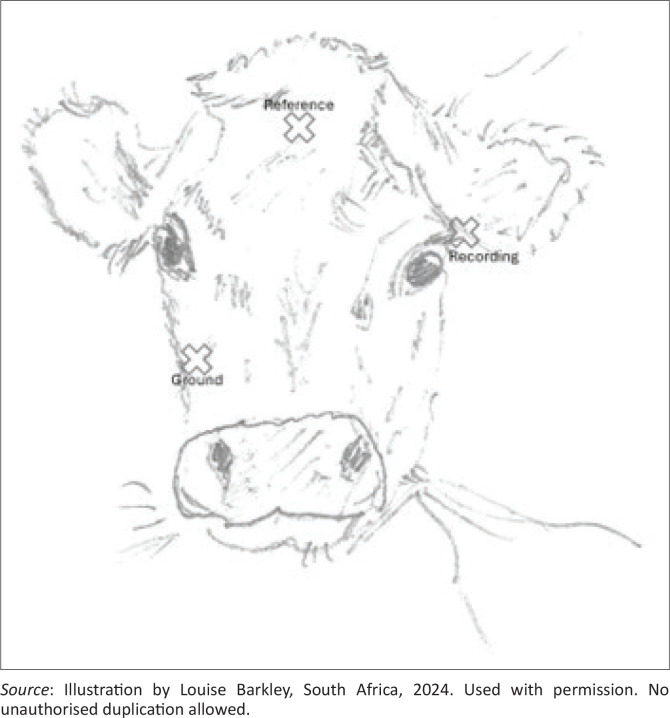
Subdermal placement of needle electrodes for brainstem auditory evoked response recordings.

Disposable subdermal single sterile needle electrodes from Rhythmlink International, measuring 13 mm in length and 0.4 mm in diameter, were inserted into the subcutaneous tissue at three standardised scalp locations by a veterinarian. The electrode needles were placed in the standard locations specific to bovine anatomy: the first was positioned at the intercornual protuberance (the midpoint between the horns and the nasal bones), serving as the reference electrode. The second electrode was placed below the stimulated ear, near the mastoid area, serving as the recording electrode, and the third electrode (ground) was placed near the nasal bones along the midline of the skull (Figure 1).

These placements are similar to those used in human BAER testing, where electrodes are typically placed on the scalp near the vertex and mastoid, which corresponds to the areas closest to the auditory pathways. However, slight modifications are necessary because of differences in anatomy between humans and cows. In cows, the placement at the intercornual protuberance and near the nasal bones takes into account the anatomical features unique to bovines, ensuring that the electrodes are positioned optimally for recording auditory evoked potentials. These adjustments are crucial for obtaining accurate and reliable BAER readings in cows. Impedance for the electrodes was maintained below 5 kΩ to ensure optimal signal quality and accurate recording.

The BAER testing was conducted using the CUB EP program (Path Medical Solutions Equipment, Germany). Stimuli design and recordings were executed with CUB firmware revision 2.7.1 (build 10993). Broadband click stimuli of 0.1 ms duration with a 24 000 Hz bandwidth were used for stimulation. The stimuli were presented monoaurally, starting with the left ear, using a rarefaction polarity. Contralateral masking was applied at a level 45 dB below the presentation stimulus in the test ear at a rate of 21.1 clicks per second. These clicks, although containing energy between 500 Hz and 8000 Hz, primarily stimulated the cochlea’s 2000 Hz–4000 Hz region, which is critical for assessing auditory thresholds. Evoked responses were collected and averaged over 1200 repetitions for each sound intensity level.

To ensure the accuracy of the stimulus intensity, the manufacturer’s correction factor was applied. Specifically, the Path Medical GmbH CUB system used for this study required a correction factor of 35 dB for click stimuli when insert earphones were used. Therefore, 105 dB SPL employed in our equipment could correspond to approximately 70 dB nHL as reported by other authors. This adjustment was necessary to accurately reflect the stimulus intensity presented to the animals, ensuring that the data obtained were comparable to other studies using similar methodologies.

This protocol was selected because it closely mirrors the standard protocols used in human BAER testing, where broadband clicks are commonly employed to evaluate auditory function, particularly within the speech frequency range (Legatt, 2013). The chosen click parameters are well established in both human and animal audiology for their reliability in eliciting clear and reproducible auditory evoked potentials. Additionally, this protocol follows the manufacturer’s recommendation for testing animals, ensuring that the setup is optimised for the specific needs of veterinary applications. The use of contralateral masking and specific click rates is consistent with practices in human audiology, where these elements help to isolate the response from the stimulated ear and reduce the potential for cross-hearing (Hall, 2007). The protocol’s effectiveness in stimulating the 2000 Hz to 4000 Hz region of the cochlea is particularly relevant, as this frequency range is crucial for understanding the auditory capabilities of both humans and animals (Gelfand, 2016). By employing this protocol, the study aligns with established practices in both human and animal BAER testing, ensuring that the data collected are comparable with existing literature and can contribute to the broader understanding of auditory function across species.

To present the acoustic stimuli, earphones (PATH Medical GmbH, Germering, Germany) were inserted into the ear canal and held in position with standard 17.8 mm diameter foam ear tips (3MTM E-A-RLINKTM 3C) to attenuate environmental noise. To ensure the correct and adequate placement of the earphones, the insertion was performed by an experienced veterinarian, carefully inserted the earphones to the appropriate depth within the ear canal, ensuring a snug fit that would prevent sound leakage. The positioning was then verified by checking for a consistent acoustic seal, which was monitored by the testing equipment to ensure optimal delivery of the stimuli. Additionally, any movement of the earphones was minimised by securing them in place throughout the testing procedure. The starting intensity was 105 dB SPL, and the intensity was decreased in 5 dB steps to estimate the auditory threshold.

### Data collection and analysis

The collected recordings underwent filtering with a high pass at 80 Hz and low pass at 2000 Hz and were analysed using MIRA software revision 2.3.2 (build 8693). The electric potentials, identified with consecutive Roman numerals, are generated by the activation of one of the subcortical components of the acoustic pathway (Legatt, 2013). Peak identification was performed by analysing the waveform morphology, which consists of a series of positive and negative deflections. Wave peaks (I–V) were manually labelled, and interpeak latencies and absolute peak amplitudes were automatically calculated by the software. Recordings were reviewed in descending order of stimulation intensity, and for each ear, the threshold intensity level was determined by excluding criteria: the disappearance of peak wave V or when the average amplitude of the remaining peaks did not exceed background electric noise levels by more than 20%. While there is a lack of universally standardised criteria for establishing auditory thresholds in cows using BAER, we selected and adhered to the approach used by Gonzalez et al. (2019) to maintain consistency and ensure that our data could be compared with existing literature. In doing so, we contributed to a growing body of research that could eventually lead to standardisation in the field.

Reliability of the measurements was ensured through repeated testing of each ear at different intensity levels to confirm consistency in wave patterns and thresholds.

### Noise measurement

Ambient noise levels were measured in the milking parlour and feedlot areas of the Hillcrest experimental farm using an XL2 sound level meter to assess the daily environmental noise levels exposure for the cows. The noise level in the milking parlour was recorded at 54.6 dB SPL, while the feedlot area registered a noise level of 27.3 dB SPL. These measurements provided important context for understanding the environmental conditions under which the auditory assessments were conducted.

### Data analysis

Descriptive statistical analysis of the BAER data was performed to assess the auditory function of Holstein cows. This included the calculation of absolute peak latencies (I–V), interpeak latencies and auditory thresholds across different sound intensities. The analysis was conducted using the Data Analysis function in Microsoft Excel 365, version 2104.

The specific steps in the data analysis process included:

- **Data preparation:** Raw data from the BAER recordings were first reviewed for consistency and accuracy. Any anomalies, such as noise artefacts or incomplete data sets, were identified and addressed before analysis.
- **Calculation of latencies:** The absolute peak latencies for waves I through V were calculated for each cow at each sound intensity level. Interpeak latencies (I–III, III–V and I–V) were also calculated to assess the neural conduction times between different points in the auditory pathway.
- **Auditory threshold determination:** Auditory thresholds were determined based on the lowest sound intensity level at which a consistent wave V response could be detected. This threshold was identified for each cow and compared across the group to assess variability.
- **Descriptive statistics:** Descriptive statistics, including mean, standard deviation and range, were calculated for peak latencies, interpeak latencies and auditory thresholds. These statistics were used to summarise the central tendency and variability of the data, providing a clear understanding of the auditory characteristics of the cows. The demographic characteristics of the sample were carefully controlled to ensure homogeneity across the study. All subjects were Holstein cows in their second lactation with normal pigmentation, from the same herd and housed under identical environmental conditions. This homogeneity in demographic factors was crucial for minimising variability in the BAER results, allowing for a more accurate assessment of auditory function across the sample. The demographic data, therefore, served primarily for descriptive purposes, ensuring that the sample was representative and that any variations in BAER results could be attributed to factors other than demographic differences.
- **Consideration of amplitude:** While the primary focus of the analysis was on latencies and thresholds, the amplitude of the waveforms was observed qualitatively. Amplitude was not analysed quantitatively because of variability across recordings and the challenges associated with ensuring consistent electrode placement and signal acquisition in large animals like cows. However, significant deviations in amplitude that might indicate abnormal auditory function were noted and considered in the overall assessment of the auditory responses.
- **Comparison with normative data:** The results were compared with existing normative data from both human and animal studies to evaluate the relevance and significance of the findings. This comparison helped to contextualise the results within the broader field of auditory research.

The use of Microsoft Excel 365 for data analysis was chosen for its accessibility and capability to handle large data sets efficiently. The statistical functions available in Excel allowed for the straightforward calculation of descriptive statistics and facilitated the comparison of data across different conditions and subjects.

### Ethical considerations

This study adhered to ethical guidelines for the use of animals in research, ensuring that all procedures were conducted with the utmost care to minimize harm and distress to the animals. Ethical approval was obtained by the Faculty of Veterinary Science, University of Pretoria, Animal Ethics Committee (reference no. NAS341/2020). The Brainstem Auditory Evoked Responses (BAER) test was non-invasive, and no pharmacological agents were used for restraint, reducing the potential for stress. Cows were kept in familiar environments and handled by experienced personnel to ensure their welfare. The study followed protocols approved by the institutional ethics committee to safeguard the animals’ well-being. Furthermore, attention was given to minimize environmental noise exposure during testing, and all efforts were made to ensure the cows’ comfort throughout the procedures. Ethical considerations also extended to the broader goal of the research, which aims to improve animal welfare by assessing the impact of noise on their auditory health, thus contributing to sustainable and humane farming practices.

## Results

The results demonstrate the values for absolute wave latencies and interpeak latencies recorded in the 10 Holstein cows (i.e. 20 ears). The BAER testing revealed consistent wave patterns across ears, with well-defined peaks representing specific auditory structures along the brainstem. Table 1 presents the detailed measurements of these latencies for the highest intensity used during testing, illustrating the auditory processing capabilities and neural transmission times within the cows. The analysis included a comparison of BAER responses between the left and right ears for each cow. No significant differences were observed in absolute wave latencies or interpeak latencies between the left and right ears, indicating that auditory function was symmetrical across both sides. This finding is consistent with previous studies in both human and animal models, where significant asymmetries are typically not expected unless there is an underlying auditory pathology.

**TABLE 1:** Absolute wave latencies and interpeak latencies at 105 dB SPL (*N* = 20).

Cattle ID name	Ear tested (side)	Absolute wave latencies (ms)				Interpeak latencies (ms)			
		I	II	III	V	I–III	III–V	II–V	I–V
Cow 1	R L	1.6 1.7	2.3 2.1	3.1 3.3	4.6 4.7	1.50 1.56	1.44 1.44	2.31 2.57	2.94 3.00
Cow 2	R L	1.6 1.5	2.3 2.1	3.1 3.2	4.7 4.7	1.44 1.69	1.63 1.50	2.44 2.57	3.07 3.19
Cow 3	R L	1.6 1.6	2.3 2.2	3.3 3.2	4.7 4.7	1.63 1.63	1.44 1.50	2.44 2.50	3.07 3.13
Cow 4	R L	1.5 1.5	2.2 2.2	3.1 3.2	4.7 4.8	1.56 1.69	1.63 1.56	2.50 2.56	3.19 3.25
Cow 5	R L	1.6 1.7	2.2 2.1	3.3 3.3	4.8 4.7	1.63 1.56	1.50 1.44	2.56 2.57	3.13 3.00
Cow 6	R L	1.6 1.5	2.2 2.1	3.2 3.2	4.7 4.8	1.57 1.69	1.50 1.62	2.50 2.69	3.07 3.31
Cow 7	R L	1.6 1.5	2.1 2.1	3.3 3.3	4.8 4.8	1.69 1.81	1.50 1.44	2.69 2.69	3.19 3.25
Cow 8	R L	1.5 1.6	2.1 2.2	3.3 3.1	4.9 4.8	1.75 1.50	1.63 1.63	2.76 2.56	3.38 3.13
Cow 9	R L	1.6 1.5	2.3 2.3	3.3 3.1	4.7 4.9	1.63 1.62	1.44 1.76	2.44 2.63	3.07 3.38
Cow 10	R L	1.4 1.4	2.1 2.1	3.2 3.0	4.7 4.6	1.75 1.56	1.50 1.56	2.57 2.50	3.25 3.12
Mean		1.56	2.18	3.21	4.74	1.62	1.53	2.55	3.16
s.d.		0.08	0.08	0.09	0.08	0.09	0.09	0.11	0.12

Note: In human BAER norms, wave I latencies typically range from 1.5 ms to 2.0 ms, wave III from 3.5 ms to 4.5 ms and wave V from 5.5 ms to 6.5 ms, with interpeak latencies (I-III, III-V and I-V) ranging between 1.6 ms and 2.2 ms, 1.8 ms and 2.5 ms and 3.9 ms and 4.5 ms, respectively (Hall, 2007; Legatt, 2013). Variations are expected because of species-specific differences.

L, left; R, right; s.d., standard deviation.

By considering the potential for lateral differences, this study confirms that the auditory responses in Holstein cows are consistent between the left and right ears, further supporting the reliability of the data collected.

The relative wave latencies (I, II, III and V) measured in the 10 cows are reported in Table 2, according to the intensity of the auditory stimulus applied (dB SPL) during the assessment. Absolute mean wave latencies (I, II, III and V) increased as the intensity of the sound stimulus decreased (Table 2). All the cows had identifiable wave V responses at 90–100 dB SPL. A wave V could be identified in nine of the cows at 85 dB SPL. No responses were obtained at lower intensities, showing 85 dB SPL to be the lowest threshold found in this group.

**TABLE 2:** Relative wave latencies at the auditory stimulus applied (*N* = 20).

Stimulus (dB SPL)	Absolute wave latencies (ms)											
	I			II			III			V		
	*n*	Mean	s.d.	*n*	Mean	s.d.	*n*	Mean	s.d.	*n*	Mean	s.d.
105	20	1.56	0.08	20	2.18	0.08	20	3.21	0.09	20	4.74	0.08
100	20	1.67	0.10	20	2.24	0.09	20	3.34	0.13	20	4.86	0.11
95	18	1.80	0.13	20	2.36	0.13	18	3.49	0.14	20	5.02	0.18
90	10	1.91	0.14	10	2.45	0.14	12	3.63	0.16	20	5.17	0.21
85	10	2.08	0.14	10	2.64	0.17	10	3.72	0.20	18	5.34	0.27
80	No wave			No wave			No wave			No wave		

Note: Human BAER wave latencies typically increase as stimulus intensity decreases. For example, at 105 dB SPL, wave V is usually around 5.5 ms to 6.0 ms, but at lower intensities like 80 dB SPL – 85 dB SPL, wave V can be delayed up to 6.5 ms (Hall, 2007). Comparisons with cow data must consider species-specific variations.

BAER, brainstem auditory evoked responses; s.d., standard deviation.

The relative interpeak latencies (I–III, III–V, II–V and I–V) measured in the 10 cows are shown in Table 3, according to the intensity of the auditory stimulus applied (dB SPL) during testing. The interpeak latencies remained similar regardless of the intensity of the stimulus (Table 3).

**TABLE 3:** Relative interpeak latencies at the auditory stimulus applied (*N* = 20).

Stimulus (dB SPL)	Interwave latencies (ms)							
	I–III		III–V		II–V		I–V	
	Mean	s.d.	Mean	s.d.	Mean	s.d.	Mean	s.d.
105	1.62	0.09	1.53	0.09	2.55	0.11	3.16	0.12
100	1.68	0.12	1.53	0.16	2.63	0.14	3.20	0.13
95	1.71	0.15	1.49	0.16	2.66	0.19	3.21	0.15
90	1.69	0.12	1.53	0.16	2.65	0.18	3.22	0.16
85	1.65	0.14	1.55	0.20	2.67	0.28	3.20	0.22

Note: Human BAER interpeak latencies are generally stable across stimulus intensities, with typical I–III latencies ranging from 1.6 ms to 2.2 ms, III–V from 1.8 ms to 2.5 ms and I–V from 3.9 ms to 4.5 ms (Legatt, 2013). Differences in cow data may reflect species-specific auditory processing.

BAER, brainstem auditory evoked responses; s.d., standard deviation.

The absolute wave latencies and interpeak latencies obtained from the normalised response according to the criterion of equivalent auditory stimulus were investigated and the results are shown in Table 4. As depicted by the standard deviation, it is clear that the results were similar across the 10 cows (20 ears).

**TABLE 4:** Wave latencies and interpeak latencies obtained for 105 dB SPL.

Wave complex	Mean (ms)	s.d. (ms)	Maximum (ms)	Minimum (ms)
I	1.56	0.08	1.70	1.40
II	2.18	0.08	2.30	2.10
III	3.21	0.09	3.30	3.00
V	4.74	0.08	4.90	4.60
I–III	1.62	0.09	1.81	1.44
III–V	1.53	0.09	1.76	1.44
II–V	2.55	0.11	2.76	2.31
I–V	3.16	0.12	3.38	2.94

s.d., standard deviation.

### Noise levels in the farm environment

The acoustic sound levels of various environmental noise sources were measured as part of this study to assess their potential impact on cow auditory health. The noise levels from the milking machine were found to range from 39.6 to 70.2 dB SPL, which is lower compared to the noise levels measured in a tandem milking parlour (72.50 dB SPL) (Pšenka et al., 2016). Additionally, the automated system during milking produced lower noise levels, around 67.92 dB SPL. In contrast, the noise level in the dirt lot (sleeping area) was recorded at 27.3 to 54.6 dB SPL, which falls within the acceptable hearing thresholds for cows. These measurements suggest that the noise levels in the farm environment are within a range that is unlikely to cause auditory damage to cows, but further investigation is needed to determine the long-term effects of chronic exposure to these noise levels.

## Discussion

In this study, we observed that the BAER thresholds in Holstein cows at 90 dB SPL, which translate to approximately 55 dB nHL, align closely with the behavioural thresholds reported by Uetake et al. (1996) for the 2000 Hz–4000 Hz range. This consistency not only reinforces the reliability of BAER as a surrogate for behavioural audiometry in cattle but also suggests that the method can provide accurate assessments of auditory function across different testing conditions and cattle populations. Behavioural audiometry, while effective, often requires more invasive procedures or relies on subjective responses, making BAER a more practical and non-invasive alternative in both research and clinical settings.

The latencies observed in our study, which range from 1.56 ms (wave I) to 4.47 ms (wave V), also fall within the range reported by Strain et al. (1989) and Arai and Matsui (2008). These studies, though conducted several decades ago, remain relevant today as they laid the groundwork for using BAER in livestock. The alignment of our findings with those of these earlier studies underscores the robustness of BAER as a diagnostic tool, even as technology and testing environments have evolved. This suggests that the auditory pathways in cattle are sufficiently similar across different breeds and environments, allowing for consistent and reliable BAER measurements.

Our study builds on the methodology established by Gonzalez-Blanco et al. (2019), whose work was instrumental in developing standardised protocols for BAER testing in cattle. By adhering closely to the protocols outlined by González et al., we ensured that our results were directly comparable to theirs, thus contributing to the establishment of normative data for BAER in bovine species. This methodological consistency is crucial for advancing the field of animal audiology, as it enables researchers to compare findings across different studies and build a cohesive body of knowledge.

Furthermore, our findings contribute to the limited body of research on the impact of environmental noise on cattle. The noise levels encountered in modern farming environments, such as those produced by machinery, tractors and automated milking systems, are known to vary widely and can reach levels that may be harmful to cattle. By establishing normative BAER data for Holstein cows, our study provides a critical reference point for future research investigating how chronic exposure to different types and levels of noise may affect the auditory health of cattle. This is particularly important given the increasing prevalence of noise pollution in agricultural settings, which has been linked to stress, reduced productivity and compromised welfare in livestock.

The importance of considering the specific characteristics of the auditory stimuli used in BAER testing cannot be overstated. In our study, we used broadband clicks, which primarily stimulate the 2000 Hz–4000 Hz frequency range. While this approach offers a reliable measure of general auditory function, it may not fully capture the cow’s sensitivity to lower-frequency noises that are more common in farm environments, such as the low hum of machinery or the sounds of other animals. Future studies should explore the use of a broader range of stimuli, including pure tones at various frequencies, to provide a more comprehensive understanding of bovine auditory health. This would allow for a more nuanced assessment of how several types of environmental noise affect cattle and help develop targeted strategies for mitigating the negative impacts of noise exposure.

Additionally, it is important to consider the implications of our findings within the broader context of animal welfare and sustainable agricultural practices. The One Health initiative, which recognises the interconnectedness of human, animal and environmental health, underscores the importance of safeguarding the auditory health of livestock as part of a holistic approach to promoting overall well-being. Ensuring that cattle are not exposed to harmful levels of noise can enhance their welfare, improve productivity and contribute to more sustainable farming practices. Our study aligns with the goals of the SDGs, particularly SDG 3 (Good Health and Well-being) and SDG 12 (Responsible Consumption and Production), by promoting practices that support the health and well-being of livestock and, by extension, the humans who depend on them.

The relevance of this study extends beyond the immediate findings on BAER thresholds and latencies. By providing normative data and validating the use of BAER as a diagnostic tool in cattle, we lay the groundwork for further research aimed at improving the welfare of farm animals. Future research could explore how BAER technology can be integrated into routine veterinary practice, particularly in assessing the auditory health of livestock exposed to high levels of environmental noise. Such research would not only advance the field of animal audiology but also contribute to the development of best practices for noise management in agricultural settings.

While this article offers significant insights into animal audiology and the auditory welfare of dairy cows, it is important to acknowledge some limitations. Despite the clear value of the results, several factors may affect the overall applicability and robustness of the results. These limitations include the small sample size and the limited direction in the field with regard to methodology and analysis. The current study significantly advances our understanding of bovine auditory health and the potential role of BAER in monitoring and protecting it. By building on the work of previous researchers applying standardised protocols and addressing these limitations in future research, we could enhance the understanding and impact of this field. Our study provides valuable preliminary data that could be used to develop normative data that can serve as a benchmark for future studies. Our results highlight the importance of ongoing research in this area and the need for a multidisciplinary approach to improving animal welfare in agricultural settings. The integration of BAER technology into veterinary practice holds promise for enhancing the health and productivity of livestock, contributing to more sustainable and humane farming practices.

### Call for action: Expanding animal audiology in South Africa

While veterinary scientists currently oversee hearing assessments in animals within the South African context, the emerging field of animal audiology presents a unique opportunity for expanding the scope of audiologists’ practice. Given that audiologists are trained in the detailed assessment of auditory function, their expertise can be invaluable in conducting diagnostic tests like BAER, which provide more precise information than the basic pass or refer screenings typically performed by veterinarians.

As animal welfare becomes an increasingly important focus in South Africa, particularly in the agricultural sector, there is a strong case for exploring the role of trained audiologists in this domain. By integrating animal audiology into their practice, audiologists could significantly contribute to improving the welfare of farm animals, enhancing productivity and supporting sustainable farming practices.

Further research and policy discussions are needed to investigate how animal audiology could be formally recognised and integrated into the professional scope of South African audiologists. This would not only align with global trends in animal welfare but also address specific challenges and opportunities within the South African agricultural landscape.

## Conclusion

This study demonstrates the utility of BAER as a reliable, non-invasive method for assessing auditory function in Holstein cows. The findings provide preliminary normative data on auditory thresholds, wave latencies and interpeak latencies, contributing significantly to the limited body of research on bovine auditory health. The consistency and reproducibility of the BAER responses across the 20 ears tested highlight the method’s effectiveness in evaluating the auditory pathway in cows.

Our results indicate that the auditory thresholds measured by BAER are comparable to those obtained through behavioural audiometry, suggesting that BAER can serve as a surrogate for more invasive or subjective methods. However, the use of broadband clicks, which primarily stimulate the 2000 Hz–4000 Hz frequency range, may not fully capture sensitivity to lower-frequency environmental noises common in farm settings, underscoring the need for future research using a broader range of stimuli.

This study not only contributes to the growing body of knowledge in animal audiology but also aligns with the broader goals of the One Health initiative and the SDGs, particularly SDG 3 (Good Health and Well-being) and SDG 12 (Responsible Consumption and Production). By safeguarding the auditory health of dairy cows, this research supports sustainable agricultural practices and animal welfare.

In conclusion, the study lays the groundwork for advancing animal audiology as a field, with implications for improving cow welfare, enhancing veterinary practice and promoting more humane and sustainable farming practices.
